# Association of serum angiopoietin-like protein 2 and epinephrine levels in metabolically healthy but obese individuals: *In vitro* and *in vivo* evidence

**DOI:** 10.3892/etm.2013.1045

**Published:** 2013-04-03

**Authors:** QING-XIN MENG, LONG WEN, XIN-YU CHEN, HUI-JU ZHONG

**Affiliations:** Department of Endocrinology, Xiangya Hospital, Central South University, Changsha, Hunan 410008, P.R. China

**Keywords:** angiopoietin-like protein 2, epinephrine, metabolically healthy but obese, insulin sensitivity, β-adrenoceptor

## Abstract

In the present study, we explored the association of serum angiopoietin-like protein 2 (ANGPTL2) levels with insulin sensitivity and serum epinephrine levels in metabolically healthy but obese (MHO) subjects. We also investigated the effects of epinephrine on ANGPTL2 expression in adipocytes *in vitro*. We examined the metabolic characteristics and serum ANGPTL2 and epinephrine levels in 100 non-diabetic obese postmenopausal women. Subjects were classified as MHO (n=25) or at-risk (n=25) based on the upper and lower quartiles of insulin sensitivity, respectively. Differentiated 3T3-L1 adipocytes were treated with increasing doses of epinephrine (10, 30 and 50 nM) in the presence or absence of phentolamine (10 *μ*M), propranolol (0.3 *μ*M), LY294002 (50 *μ*M) or protein kinase A inhibitor fragment 6–22 amide (PKAI, 1 mM) for 24 h. We observed that serum ANGPTL2 levels were negatively correlated with insulin sensitivity (r=−0.23, P=0.021) and serum epinephrine level (r=−0.62, P<0.001) in the study subjects, with the MHO subjects displaying significantly lower serum ANGPTL2 and higher serum epinephrine levels than the at-risk subjects. Epinephrine reduced the ANGPTL2 mRNA and protein levels in differentiated 3T3-L1 adipocytes in a dose-dependent manner. Propranolol and PKAI were able to eliminate this reduction in ANGPTL2 levels whereas phentolamine and LY294002 were not. The *in vitro* findings indicated that epinephrine decreased ANGPTL expression at the mRNA and protein levels via the β-adrenoceptors and the PKA signaling pathway. This study suggests that β-receptor activation helps to maintain the metabolic profile of MHO individuals and prevent type 2 diabetes mellitus (T2DM) by decreasing serum ANGPTL2 levels.

## Introduction

Obesity is a pandemic medical and social problem that is associated with several adverse health outcomes, including type 2 diabetes mellitus (T2DM), hypertension, dyslipidemia, cardiovascular disease and cancer (1,2), all of which result in increased mortality. Notably, 20–30% of the adult obese population, referred to as metabolically healthy but obese (MHO) individuals, have been identified who, despite having excessive adiposity, are relatively insulin sensitive and have a favorable cardiovascular risk profile (3,4). Several studies have examined characteristics associated with the protective profile of MHO individuals (5,6). Brochu *et al* reported that an early age of obesity onset and low amounts of visceral adipose tissue had protective effects on MHO postmenopausal women (5). Karelis *et al* reported that a lower inflammation state, as attested by low C-reactive protein (CRP) levels, may play a role in the protective profile of MHO postmenopausal women (6). Dai *et al* reported a positive association between plasma epinephrine level and insulin sensitivity in MHO individuals (7). In multiple regression analysis, CRP, epinephrine, triglycerides and the lean body mass index were identified as independent predictors of glucose disposal, collectively explaining 39.4% of the variance in insulin sensitivity.

Angiopoietin-like proteins (ANGPTLs), which are structurally similar to angiopoietins, are characterized by a coiled-coil domain in the N-terminus and a fibrinogen-like domain in the C-terminus (8). ANGPTL2, a member of the ANGPTL family, has been shown to be expressed abundantly in adipose tissues and is reportedly a key mediator linking obesity to adipose tissue inflammation and systemic insulin resistance in mice and humans (9,10). However, the association of serum ANGPTL2 levels with MHO phenotype has not yet been investigated. In the present study, we explored the association of serum ANGPTL2 levels with insulin sensitivity and serum epinephrine levels in non-diabetic obese postmenopausal women, and investigated the effects of epinephrine on ANGPTL2 expression in adipocytes *in vitro.*

## Materials and methods

### Subjects

A total of 100 non-diabetic obese postmenopausal women aged between 50 and 76 years old were enrolled in this study using the following criteria: i) body mass index (BMI) >27 kg/m^2^; ii) cessation of menstruation for >1 year and a follicle-stimulating hormone level of ≥30 U/l; iii) sedentary (<2 h of structured exercise per week); iv) nonsmoker; v) low to moderate alcohol consumer (fewer than two drinks per day); vi) free of known inflammatory disease; and vii) no use of hormone replacement therapy. On physical examination or biological testing, all participants had no history or evidence of the following: i) cardiovascular disease, peripheral vascular disease or stroke; ii) diabetes 2-h plasma glucose <11.0 mmol/l after a 75-g oral glucose tolerance test; iii) orthopedic limitations; iv) body weight fluctuation within 2 kg in the last 6 months; v) thyroid or pituitary disease; vi) infection according to medical questionnaire examination and complete blood count; and vii) medication that may affect cardiovascular function and/or metabolism. Informed consent was obtained from all subjects prior to the start of the study. After a 4-week period of weight stabilization, patients underwent a 3-hour hyperinsulinemic-euglycemic (HE) clamp. A blood draw was performed for determination of a fasting lipid profile and analyses of insulin and glucose. Body composition was assessed by dual-energy X-ray absorptiometry a few days after the HE clamp. This study was approved by the Ethics Committee of Xiangya Hospital, Changsha, China.

### Identification of MHO and at-risk subjects

As previously described by Karelis *et al*(6), we identified MHO and at-risk subjects by dividing the entire cohort of 100 patients into quartiles based on glucose disposal rates (M values/FFM). Glucose disposal (M_(clamp)_) was calculated as the mean rate of glucose infusion measured during the last 30 minutes of the clamp (steady-state) and is expressed as milligrams per minute per kilogram body weight or as milligrams per minute per kilogram fat-free mass (FFM). Women with M/FFM values in the upper quartile (M≥12.84; n=25) were classified as having high insulin sensitivity and placed in the MHO group, whereas women with M/FFM values in the lower quartile (M≤9.05; n=25) were classified as low insulin sensitivity and categorized as at-risk subjects. The at-risk group was defined as a group that presents metabolic abnormalities (i.e. insulin resistance and dyslipidemia), which may be associated with an increased risk of T2DM and/or cardiovascular disease.

### Laboratory analysis

Prior to the HE clamp, blood samples were drawn from subjects who had been resting quietly for 30 min in a recumbent position following the insertion of a venous catheter. The subjects refrained from eating, using tobacco, or drinking coffee or tea for at least 4 h prior to veni-puncture. The procedure room was kept quiet and comfortable at a temperature of 23–24° C. Serum high-sensitivity CRP (hsCRP) and β-1 anti-trypsin were assessed with ELISA kits from antibodies-online.com (Atlanta, GA, USA) and Bethyl Laboratories, Inc. (Montgomery, TX, USA), respectively. Basal serum epinephrine, norepinephrine and dopamine levels were assessed with a 3-CAT RIA kit from Rocky Mountain Diagnostics, Inc. (Colorado Springs, CO, USA).

### Cell line and reagents

The 3T3-L1 cell line was purchased from the American Type Culture Collection (Manassas, VA, USA). Anti-ANGPTL2 (sc-107143) antibody and anti-β-actin (sc-130656) antibody were purchased from Santa Cruz Biotechnology, Inc. (Santa Cruz, CA, USA). All secondary antibodies were purchased from Jackson ImmunoResearch Laboratories (West Grove, PA, USA). Epinephrine bitartrate, phentolamine, propranolol, protein kinase A inhibitor fragment 6–22 amide (PKAI), LY294002 and all chemicals of reagent grade were purchased from Sigma (St. Louis, MO, USA).

### Cell culture and treatment

3T3-L1 cells were cultured in DMEM with 10% FCS, 10 U/ml penicillin, 10 *μ*g/ml streptomycin and 0.5 *μ*g/ml amphotericin B for 10 days in 5% CO_2_ at 37°C. The cells were differentiated into mature adipocytes in medium supplemented with insulin (1.7 M), IBMX (1 *μ*M) and dexamethasone (25 pM) for 2 days. After culture in medium with insulin (1.7 *μ*M) for an additional 6 days, the adipocytes were used for experiments within 7–11 days. At the time of the experiments >90% of the cells had accumulated lipid droplets. 3T3-L1 cells were treated with epinephrine (10, 30, or 50 nM) in the presence or absence of phentolamine (10 *μ*M), propranolol (0.3 *μ*M), LY294002 (50 *μ*M) or PKAI (1 mM) for 24 h.

### Western blot analysis

Protein was extracted with lysis buffer containing 150 mM NaCl, 2% Triton, 0.1% SDS, 50 mM Tris pH 8.0 and 10% protease inhibitor cocktail (Sigma) and stored at −20° C. Equal amounts of protein (25 *μ*g) for each sample were loaded into pre-cast 7.5% Mini Protean TGX gels (Bio-Rad, Hercules, CA, USA) and separated by electrophoresis for 50 min at 200 V. The separated proteins were transferred to a PVDF transfer membrane (Amersham Biosciences/GE Healthcare, Piscataway, NJ, USA) for 55 min at 100 V. The membranes were incubated for 1 h with a 1/500 dilution of anti-ANGPTL2 and 1/1000 dilution of anti-β-actin antibody, and then washed and revealed using secondary antibodies with horseradish peroxidase conjugate (1/5000, 1 h). Peroxidase was revealed with an GE Healthcare ECL kit. Proteins were quantified before being loaded onto the gel and equal loading of protein was verified by Ponceau coloration.

### Real-time quantitative reverse transcription (RT)-PCR

RNA was prepared from 3T3-L1 adipocytes using TRIzol reagent followed by purification with TURBO DNA-free system (Ambion, Austin, TX, USA). The cDNAs were synthesized using SuperScript II reverse transcriptase (Invitrogen Life Technologies, Carlsbad, CA, USA). Real-time quantitative PCR was performed on the LightCycler thermal cycler system (Roche Diagnostics, Indianapolis, IN, USA) using a SYBR-Green I kit (Roche) according to the manufacturer’s instructions. The results were normalized against the level of the housekeeping gene glyceraldehyde-3-phosphate dehydrogenase (GAPDH) in the same sample. The primers used were as follows: for ANGPTL2, 5′-GGAGGTTGGACTGTCATCCAGAG-3′ (forward) and 5′-GCCTTGGTTCGTCAGCCAGTA-3′ (reverse); for GAPDH, 5′-ATTCAACGGCACAGTCAAGG-3′ (forward) and 5′-TGTTAGTGGGGTCTCGCTCC-3′ (reverse). Each experiment was repeated twice in triplicate.

### Statistical analysis

All continuous variable values were expressed as mean ± standard deviation. Comparisons of means between two groups were performed using a Student’s t-test upon test of normality and equality of variances. Comparisons of means among multiple groups were performed with one-way ANOVA followed by post hoc pairwise comparisons using the least significant difference method. Discrete variables were compared with Chi-square tests. A stepwise multi-linear regression model determined which variables explained unique variance in glucose disposal values. Statistical analyses were performed with SPSS for Windows 13.0 (SPSS, Chicago, IL, USA). P<0.05 was considered to indicate a statistically significant result.

## Results

By design [MHO, upper quartile of insulin sensitivity (IS) vs. at-risk, lower quartile of IS], the two groups were significantly different in absolute and relative levels of glucose disposal rates and insulin sensitivity (IS_(clamp)_; P<0.01). As shown in Table I, the MHO and the at-risk groups of obese postmenopausal women were comparable in age, BMI, fat mass index and waist circumference. While there were no significant group differences in total cholesterol, LDL-cholesterol and resting systolic and diastolic pressure, the MHO group showed higher levels of epinephrine and HDL-cholesterol and lower levels of triglycerides than the at-risk group (P<0.01). In addition, the MHO group showed significantly lower levels of ANGPTL2, hsCRP and α-1 anti-trypsin than the at-risk group (P<0.01).

Statistical analyses were performed for the entire cohort (n=100) of non-diabetic obese postmenopausal women for stepwise multi-linear regression analysis and correlation analyses. As shown in Table II, Pearsons’ correlation analyses showed that the serum ANGPTL level was negatively correlated with the glucose disposal rate [M_(clamp)_ and M/FFM_(clamp)_], insulin sensitivity [IS_(clamp)_], and the serum epinephrine level. No statistically significant correlation was noted between the serum ANGPTL2 level and the serum hsCRP or α-1 anti-trypsin level.

Multivariate regression analysis showed that among all variables listed in Table I, with the exception of the serum epinephrine level, hsCRP, ANGPTL2, triglycerides and lean body mass index were independent predictors of glucose disposal, collectively explaining 41.2% of the variance (P<0.05; Table IIIA). However, following additional adjustment for the serum epinephrine level, ANGPTL2 was no longer an independent predictor of glucose disposal (Table IIIB), suggesting that the serum epinephrine level accounted for the variances caused by the serum ANGPTL2 level in glucose disposal.

**Figure 1:**
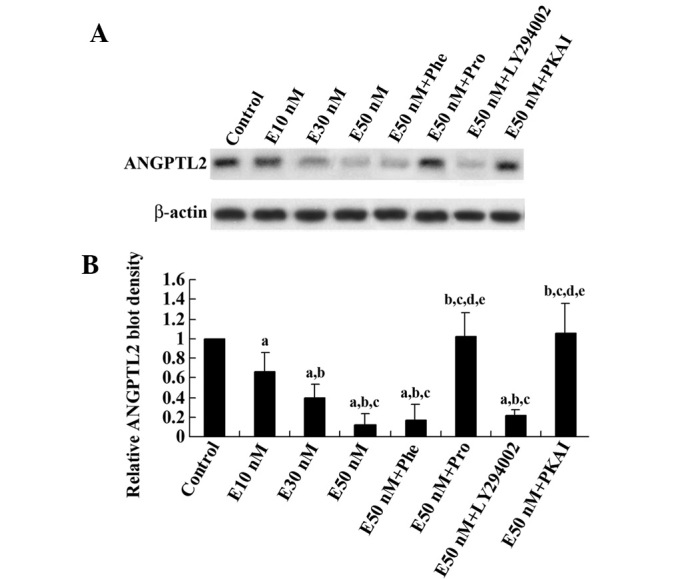
Western blot analysis of the effect of epinephrine on the protein level of angiopoietin-like protein 2 (ANGPTL2) in differentiated 3T3-L1 adipocytes. (A) Differentiated 3T3-L1 cells were treated with epinephrine in different concentrations (E10, 30, or 50 nM) for 24 h in the presence or absence of phentolamine (Phe, 10 *μ*M), propranolol (Pro, 0.3 *μ*M), LY294002 (50 *μ*M) or protein kinase A inhibitor fragment 6–22 amide (PKAI, 1 mM). Cell lysates were subject to western blot analyses for ANGPTL2 expression. Lysates from untreated cells were used as a control. β-Actin blotting was used as a loading control. (B) ANGPTL2 and β-actin blots were measured by densitometry. The density of the ANGPTL2 blot was normalized against that of β-actin to obtain a relative density, which was expressed as fold change to the relative blot density of the control (designated as 1). ^a^P<0.05 compared with untreated control cells; ^b^P<0.05 compared with E10 nM; ^c^P<0.05 compared with E30 nM; ^d^P<0.05 compared with E50; ^e^P<0.05 compared with E50 nM+Phe (10 *μ*M).

**Figure 2:**
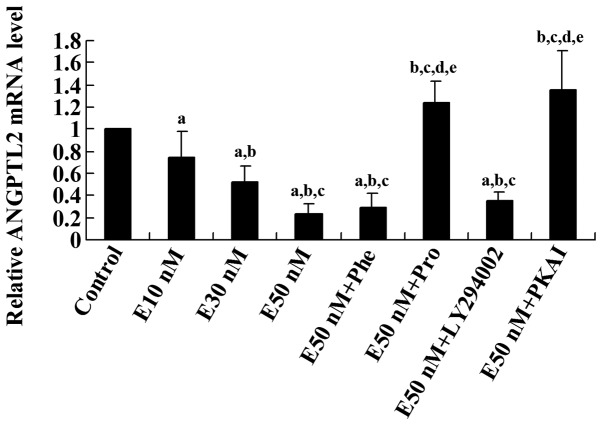
Real-time reverse transcription (RT)-PCR analysis of the effect of epinephrine on the mRNA level of angiopoietin-like protein 2 (ANGPTL2) in differentiated 3T3-L1 adipocytes. Differentiated 3T3-L1 cells were treated with epinephrine in different concentrations (E10, 30, or 50 nM) for 24 h in the presence or absence of phentolamine (Phe, 10 *μ*M), propranolol (Pro, 0.3 *μ*M), LY294002 (50 *μ*M) or protein kinase A inhibitor fragment 6–22 amide (PKAI, 1 mM) for 24 h. The ANGPTl2 mRNA level of treated cells was shown as fold change to that of the untreated control cells (designated as 1). ^a^P<0.05, compared with untreated control cells; ^b^P<0.05, compared with E10 nM; ^c^P<0.05, compared with E30 nM; ^d^P<0.05, compared with E50; ^e^P<0.05, compared with E50 nM+Phe (10 *μ*M).

Based on the *in vivo* data, we hypothesized that there was a causal relationship between serum epinephrine and ANGPTL2 levels. As ANGPTL2 is primarily secreted by adipose tissue (9), we further explored the effect of epinephrine on ANGPTL2 expression in 3T3-L1 adipocyte cells *in vitro*. As shown in Fig. 1, epinephrine reduced the ANGPTL2 protein level in differentiated 3T3-L1 adipocytes in a concentration-dependent manner. This effect was eliminated by the β-adrenoceptor blocker propranolol and PKAI, but not by the β-adrenoceptor blocker phentolamine or the phosphatidylinositol 3-kinase (PI3K) inhibitor LY294002. Real-time RT-PCR showed that the ANGPTL2 mRNA level was decreased by epinephrine in a concentration-dependent manner, which was eliminated by propranolol or PKAI, but not by phentolamine or LY294002 (Fig. 2). These results suggest that epinephrine reduces ANGPTL expression at both the mRNA and protein levels through β-adrenoceptors and the PKA signaling pathway.

## Discussion

MHO individuals are insulin sensitive, normotensive and have normal lipid profiles, despite having excessive adiposity (11). Several studies have reported the association of metabolic and inflammatory characteristics with the protective profile of the MHO individual (5,9,12). ANGPTL2 is a key adipocyte-derived inflammatory mediator linking obesity to systemic insulin resistance (9). Doi *et al* reported that elevated serum ANGPTL2 levels were positively associated with the development of T2DM in a general population, independent of other risk factors including hsCRP levels (8). In agreement with the previous reports, our study showed that the serum ANGPTL2 level was negatively correlated with the glucose disposal rate and insulin sensitivity, but not with the serum hsCRP and α-1 anti-trypsin levels in the study subjects, with the MHO subjects displaying significantly lower serum ANGPTL2 levels than the at-risk subjects (P<0.05).

The serum epinephrine and ANGPTL2 levels showed a strong negative correlation and multivariate regression analysis suggested a causal relationship between the serum epinephrine and ANGPTL2 levels. As ANGPTL2 is primarily secreted by adipose tissue in the human body, in subsequent *in vitro* experiments, we employed differentiated 3T3-L1 adipocytes as a cell model, which has been used in a previous adipocyte study (9). To examine the effects of epinephrine on ANGPTL2 expression, we treated 3T3-L1 adipocytes with low concentrations of epinephrine in small increments (10, 30 and 50 nM) for 24 h. This was due to: i) the difference in serum epinephrine levels between the MHO group and the at-risk group was ∼3-fold; and ii) the effects of circulating epinephrine on insulin sensitivity was expected to be chronic in the human body.

Despite a similar potency for α- and β-adrenoceptors, epinephrine stimulates β-receptors (particularly β2-receptors) to a greater extent than norepinephrine (13). In agreement with a previous study, our *in vitro* experiments in the present study showed that a β-, but not α-receptor blocker, completely eliminated the inhibitory effects of epinephrine on ANGPTL2 expression in adipocytes, suggesting that epinephrine increased ANGPTL2 expression via the β-receptors (13). Since serum ANGPTL2 leves links obesity with systemic insulin resistance and is positively associated with the development of T2DM (8), our results suggest that β-receptor activation helps to maintain the metabolic profile of MHO and prevent T2DM by decreasing serum ANGPTL2 levels. Although the acute effect of pharmacological doses of epinephrine is to increase blood glucose and diminish insulin sensitivity (13), the long-term effect of endogenous epinephrine is reportedly protection against hyperglycemia and insulin insensitivity in a hig-fat-diet-induced obesity mouse model (14). Epinephrine is able to stimulate intracellular AMP-activated protein kinase (AMPK) through β-receptors, which may lead to improved insulin sensitivity (15). However, the chronic use of β-blockers decreases insulin sensitivity in humans (16). Thus, chronic stimulation of β-receptors by an elevated serum level of epinephrine may increase insulin sensitivity. Our study suggests that decreasing ANGTPL2 expression through the β-receptors is one of the mechanisms underlying the protective effects of epinephrine on MHO subjects. Further studies are required: i) to define which β-receptor subtypes are involved in the effects of epinephrine on ANGPTL2 expression in adipocytes; and ii) to elaborate the underlying molecular mechanisms.

There are several limitations to our *in vivo* study. Firstly, our cohort consisted only of non-diabetic sedentary obese postmenopausal women. Therefore, our findings are limited to this population. Secondly, we used a cross-sectional approach, which does not allow us to draw any causal connection among serum ANGPTL2 and epinephrine levels and insulin sensitivity in MHO subjects. However, our *in vitro* study provided evidence for a causal relationship between serum ANGPTL2 and epinephrine levels, which compensated for the deficiencies in the *in vivo* study.

In conclusion, our *in vivo* findings show that the serum ANGPTL2 level is negatively associated with insulin sensitivity and the serum epinephrine level in non-diabetic obese postmenopausal women, with MHO subjects displaying significantly lower serum ANGPTL2 and higher serum epinephrine levels than at-risk subjects. Our *in vitro* findings indicate that epinephrine decreases ANGPTL expression at the mRNA and protein levels via β-adrenoceptors and the PKA signaling pathway. This study suggests that β-receptor activation helps to maintain the metabolic profile of MHO and prevent T2DM by decreasing serum ANGPTL2 levels.

## Figures and Tables

**Table I:** Characteristics of MHO and at-risk subjects.

Characteristics	MHO (n=25)	At-risk (n=25)
Physical characteristics		
Age (years)	59.5±7.3	60.8±6.1
Age group (years), n (%)		
<50	4 (16)	4 (16)
50–59	7 (28)	9 (36)
60–69	10 (40)	9 (36)
≥70	4 (16)	3 (12)
BMI (kg/m^2^)	33.1±4.5	35.2±3.9
Fat mass index (kg/m^2^)	15.9±3.6	15.7±3.1
Lean body mass index (kg/m^2^)	16.2±2.3^a^	19.3±2.6
Waist circumference (cm)	97.2±9.3	101.8±9.5
Metabolic characteristics		
Total cholesterol (mmol/l)	5.9±1.1	5.7±1.4
LDL-cholesterol (mmol/l)	3.8±0.9	3.9±0.7
HDL-cholesterol (mmol/l)	1.9±0.3^a^	1.4±0.4
Triglycerides (mmol/l)	1.3±0.7^a^	2.5±1.3
Systolic blood pressure (mmHg)	121.3±18.5	120.0±16.5
Diastolic blood pressure (mmHg)	79.7±9.2	80.2±8.9
Insulin sensitivity index		
Fasting glucose (mmol/l)	4.6±1.3	5.4±0.9
Fasting insulin (*μ*U/ml)	10.9±4.1^a^	19.8±6.1
HOMA-IR	2.4±1.2^a^	4.4±1.7
IS (clamp)	304.3±75.9^a^	166.5±47.0
M (clamp) (mg/min/kg)	8.6±1.3^a^	4.3±0.9
M/FFM (clamp) (mg/min/kg FFM)	15.9±2.7^a^	7.3±1.5
Inflammation markers		
hsCRP (mg/l)	2.2±2.3^a^	5.4±4.7
α-1 anti-trypsin (g/l)	1.5±0.2^a^	1.9±0.3
ANGPTL2 (ng/ml)	2.9±0.8^a^	4.2±1.3
Serum catecholamines		
Epinephrine (pg/ml)	81±24^a^	32±19
Norepinephrine (pg/ml)	335±42	319±30
Dopamine (pg/ml)	56±23	60±32

For continuous variables, with the exception of age, all values were expressed as mean ± SD. Independent Student’s t-tests were performed to compare means between the groups. For categorical variables, all values were expressed as n (%) and comparisons were performed with Chi-square tests. HOMA-IR, homeostasis model assessment for insulin resistance; FFM, fat free mass; hsCRP, high-sensitivity C-reactive protein; ANGPTL2, angiopoietin-like protein 2; MHO, metabolically healthy but obese; BMI, body mass index; LDL, low-density lipoprotein; HDL, high-density lipoprotein; IS, insulin sensitivity; M, glucose disposal.

a P<0.05 compared with the at-risk group.

**Table II:** Correlation of plasma epinephrine level with glucose disposal rate and blood lipid and inflammation marker levels.

Variable	M_(clamp)_ (mg/min/kg)	M/FFM_(clamp)_ (mg/min/kg FFM)	IS_(clamp)_	HDL cholesterol (mmol/l)	Triglycerides (mmol/l)	hsCRP (mg/l)	α-1 Anti-trypsin (g/l)	Epinephrine (pg/ml)
ANGPTL2 (ng/ml)	r=−0.25	r=−0.27	r=−0.23	r=−0.21	r=0.22	r=0.18	r=0.17	r=−0.62
	P=0.015^a^	P=0.008^a^	P=0.021^a^	P=0.028^a^	P=0.024^a^	P=0.077	P=0.095	P<0.001^a^

Pearson’s correlation analyses were performed using data from the entire cohort (n=100) of obese postmenopausal women. Glucose disposal rate is represented by M_(clamp)_ and M/FFM_(clamp)._ Insulin sensitivity is represented by IS_(clamp)._ ANGPTL2, angiopoietin-like protein 2; M, total mass; FFM, fat free mass; IS, insulin sensitivity; HDL, high-density lipoprotein; hsCRP, high-sensitivity C-reactive protein.

a P<0.05.

**Table III:** Multivariate regression analysis of independent predictors of glucose disposal in obese postmenopausal women.

A, Without adjustment for plasma epinephrine				

Variable	Partial r^2^	Total r^2^	β Coefficient	P-Value

Glucose disposal (mg/min/kg)				
hsCRP	0.185	0.185	−0.267	0.011
ANGPTL2	0.149	0.334	−0.243	0.015
Triglycerides	0.048	0.382	−0.216	0.016
Lean body mass index	0.030	0.412	−0.202	0.040

B, With adjustment for plasma epinephrine				

Variable	Partial r^2^	Total r^2^	β Coefficient	P-Value

Glucose disposal (mg/min/kg)				
HsCRP	0.176	0.176	−0.262	0.017
Epinephrine	0.172	0.348	0.259	0.010
Triglycerides	0.037	0.385	−0.183	0.021
Lean body mass index	0.020	0.405	−0.174	0.046

Stepwise multi-linear regression analysis was performed using data from the entire cohort (n=100) of obese postmenopausal women. ANGPTL2, angiopoietin-like protein 2; hsCRP, high-sensitivity C-reactive protein.

## References

[1] Eckel RH, Grundy SM, Zimmet PZ (2005). The metabolic syndrome. Lancet.

[2] Mokdad AH, Ford ES, Bowman BA, Dietz WH, Vinicor F, Bales VS, Marks JS (2003). Prevalence of obesity, diabetes, and obesity-related health risk factors, 2001. JAMA.

[3] Ruderman NB, Schneider SH, Berchtold P (1981). The ‘metabolically-obese,’ normal-weight individual. Am J Clin Nutr.

[4] Succurro E, Marini MA, Frontoni S (2008). Insulin secretion in metabolically obese, but normal weight, and in metabolically healthy but obese individuals. Obesity (Silver Spring).

[5] Brochu M, Tchernof A, Dionne IJ, Sites CK, Eltabbakh GH, Sims EA, Poehlman ET (2001). What are the physical characteristics associated with a normal metabolic profile despite a high level of obesity in postmenopausal women?. J Clin Endocrinol Metab.

[6] Karelis AD, Faraj M, Bastard JP, St-Pierre DH, Brochu M, Prud’homme D, Rabasa-Lhoret R (2005). The metabolically healthy but obese individual presents a favorable inflammation profile. J Clin Endocrinol Metab.

[7] Dai XP, Liu ZQ, Xu LY, Gong ZC, Huang Q, Dong M, Huang X (2012). Association of plasma epinephrine level with insulin sensitivity in metabolically healthy but obese individuals. Auton Neurosci.

[8] Doi Y, Ninomiya T, Hirakawa Y (2013). Angiopoietin-like protein 2 and risk of type 2 diabetes in a general Japanese population: the Hisayama study. Diabetes Care.

[9] Tabata M, Kadomatsu T, Fukuhara S (2009). Angiopoietin-like protein 2 promotes chronic adipose tissue inflammation and obesity-related systemic insulin resistance. Cell Metab.

[10] Okada T, Tsukano H, Endo M (2010). Synoviocyte-derived angiopoietin-like protein 2 contributes to synovial chronic inflammation in rheumatoid arthritis. Am J Pathol.

[11] Wildman RP, Muntner P, Reynolds K, McGinn AP, Rajpathak S, Wylie-Rosett J, Sowers MR (2008). The obese without cardio-metabolic risk factor clustering and the normal weight with cardiometabolic risk factor clustering: prevalence and correlates of 2 phenotypes among the US population (NHANES 1999–2004). Arch Intern Med.

[12] Romano M, Guagnano MT, Pacini G (2003). Association of inflammation markers with impaired insulin sensitivity and coagulative activation in obese healthy women. J Clin Endocrinol Metab.

[13] Westfall TC, Westfall DP, Brunton LL (2006). Neurotransmission: The autonomic and somatic motor nervous systems. Goodman and Gilman’s The Pharmacological Basis of Therapeutics.

[14] Ziegler MG, Milic M, Sun P (2011). Endogenous epinephrine protects against obesity induced insulin resistance. Auton Neurosci.

[15] Steinberg GR, Jørgensen SB (2007). The AMP-activated protein kinase: role in regulation of skeletal muscle metabolism and insulin sensitivity. Mini Rev Med Chem.

[16] Sharma AM, Pischon T, Hardt S, Kunz I, Luft FC (2001). Hypothesis: Beta-adrenergic receptor blockers and weight gain: A systematic analysis. Hypertension.

